# Content validation of a self-report daily diary in patients with sickle cell disease

**DOI:** 10.1186/s41687-021-00337-7

**Published:** 2021-07-27

**Authors:** Michelle K. White, Cory Saucier, Miranda Bailey, Denise D’Alessio, April Foster, Danielle St. Pierre, Kimberly Raymond

**Affiliations:** 1 Optum, 1301 Atwood Ave, Suite 311N, Johnston, RI USA; 2 Novartis Pharmaceutical Corporation, One Health Plaza, East Hanover, NJ USA; 3 Formerly of Optum, 1301 Atwood Ave, Suite 311N, Johnston, RI USA

## Abstract

**Background:**

Sickle Cell Disease (SCD) is a genetic progressive vascular disease that impacts patients overall health and quality of life. Sickle-cell pain crises (SCPCs) are a hallmark clinical presentation of SCD and have been associated with increased morbidity and mortality. The Sickle Cell Pain Diary- Self Report (SCPD-S) was developed as a daily patient-reported outcome (PRO) measure primarily intended to capture the frequency and severity of SCD-related pain during and outside of a SCPC. The SCPD-S also examines the impact of the pain associated with an SCPC on other health-related quality of life concepts. The objective of this study was to investigate the content validity of the SCPD-S.

**Methods:**

The content validation testing included 18 in-depth hybrid concept elicitation and cognitive debriefing interviews conducted with SCD patients in the US aged 12 years and older. Interviewers used a semi-structured interview guide and a think-aloud approach for the cognitive debriefing portion. All interviews were recorded, transcribed, coded and analyzed.

**Results:**

Eighteen interviews across two rounds were conducted. Round 1 hybrid interviews (*n* = 12) resulted in the expansion of the SCPD-S from 13 to 19 items. Items on the impact of an SCPC on social and recreational activities, sleep, and emotional well-being were added. Five items were significantly revised, as were three response choice sets. Round 2 hybrid interviews (*n* = 6) confirmed the comprehensiveness of the revised diary, understandability of the wording, and appropriateness of the recall period and response sets. Saturation analyses specific to concept elicitation revealed that no additional interviews were needed.

**Conclusions:**

This study provided evidence to support the content validity of the SCPD-S, a self-report daily diary. Data gathered during patient interviews indicated that the SCPD-S is a fit for purpose measure of SCD and SCPC-related pain frequency and severity and the impact of this pain on other health-related quality of life concepts including fatigue and emotional health. The numerous changes to the SCPD-S as a result of the study findings highlight the importance of the content validation process when developing a PRO measure.

## Introduction

Sickle cell disease (SCD) is a genetic vascular disease that affects approximately 100,000 children and adults in the US. SCD imposes a significant burden on patients, impairing their ability to function, which can lead to an inability to maintain a consistent work or school schedule, keep social engagements, and participate fully in family life [1, 2]. In the United States, SCD primarily affects African-Americans: 1 out of every 365 African-Americans are born with SCD [3, 4]. Clinical features of SCD include pallor, jaundice, anemia, and recurrent episodes of severe pain, known as sickle cell pain crises (SCPCs; also referred to as vaso-occlusive crises (VOCs)) [5, 6].

SCPCs are characterized by acute, often debilitating pain that prevents normal functioning, and have been associated with an increased risk of morbidity and mortality. SCPCs can lead to life-threatening emergencies such as acute chest syndrome, and stroke [7–10]. In addition to the acute effects of SCPCs, the cumulative effects of vaso-occlusion, with or without pain, can lead to irreversible damage to bones, joints, and organs; in particular, damage to the spleen, liver, brain, lungs, and kidneys occurs over time. This damage compounds disease-related pain while increasing morbidity and mortality [1, 7, 10, 11]. SCPCs can be triggered by physiological, environmental, nutritional, and psychosocial issues and are the primary cause of healthcare utilization in patients with SCD [1, 5, 9, 12]. The duration of a SCPC is variable, and can range from several hours to more than a week [13].

As described in the US Food and Drug Administration’s *Voice of the Patient* Sickle Cell Disease report*,* patients rated pain as the most burdensome symptom they experience [5], underscoring the way in which SCPCs adversely affect their lives. Patients of all ages described a variety of ways in which SCPCs pain negatively impacted family, work/school, and emotional health, and also discussed how SCPC pain can worsen other problematic symptoms such as fatigue, insomnia, difficulty concentrating, and mobility [5, 14]. Given their far-reaching impacts, it is necessary to determine the best way to accurately assess the frequency, severity, and impact of SCPCs on patients’ lives.

Studies often rely on acute or emergency medical visits as a way to measure SCPC-related outcomes in the SCD population [15, 16]. These methods provide an understanding of healthcare resource utilization. But, they do not incorporate the breadth and depth of patients’ experience of the burden of SCD, and fail to adequately capture daily variations of pain and other health-related quality of life concepts. Evaluating healthcare resource utilization data alone underestimates the burden of SCD because patients who avoid seeking care outside the home are not represented [17, 18]. A previous study found that individuals with SCD experienced SCPCs on 13.5% of recorded days, but only sought out clinical care on 3.5% of days over a 6-month time period, emphasizing the potential shortcomings of measuring SCPCs through medical visits alone [18].

SCPCs can be assessed through patient self-report. However, identifying the optimal way in which to collect such patient-reported outcome (PRO) data can be challenging. A retrospective format, such as that used to assess chronic pain, asks patients to report on their pain over a one-week, 1 month, or longer recall period. Retrospective formats with longer recall periods, such as 7-day or longer, may be problematic for assessing sporadic events such as SCPCs because such longer recall periods can “wash out” the severity of pain and impacts when averaged over time. Using a shorter recall period, such as “in the past 24 hours,” may be more appropriate to measure sporadic events.

The Sickle Cell Pain Diary-Self Report (SCPD-S) was developed as a self-reported PRO measure that addresses these gaps and accurately captures SCD-related concepts on a daily basis. The SCPD-S was designed to be completed by patients with SCD aged 12 and older and is intended to be administered electronically. The purpose of this study was to evaluate the content validity of the SCPD-S using two rounds of qualitative hybrid concept elicitation (CE) and cognitive debriefing (CD) individual interview methods.

## Methods

### Origin of the SCPD-S

A draft version of the SCPD-S (v.01) was developed using a multifaceted approach including a review of the literature, PRO expert input, feedback collected during a day-long patient advisory board meeting that also included clinicians, and individual discussions with clinicians. The SCPD-S v.01 included 13 items related to daily pain severity and duration, fatigue, medication use, and the impact of SCD on work/ school, daily activities, and sleep.

### Patient inclusion criteria, recruitment, and consent

Patients eligible to participate in the study were at least 12 years of age, had a self-reported diagnosis of SCD, experienced at least one SCPC in the 12 months prior to screening, and were willing and able to participate in a 90 min interview in English either in-person or via telephone. Patients were recruited by a company that specializes in recruitment for healthcare research using their proprietary patient database. Sampling procedures were designed to enroll patients diverse in demographic characteristics such as age and education level. Interested patients were directed to a brief online screening survey to provide initial confirmation of their eligibility. Telephone confirmation of eligibility was conducted prior to scheduling an interview. Patients provided written informed consent once enrolled. All study materials were approved by the New England Independent Review Board.

### Design and procedure

**Fig. 1:**
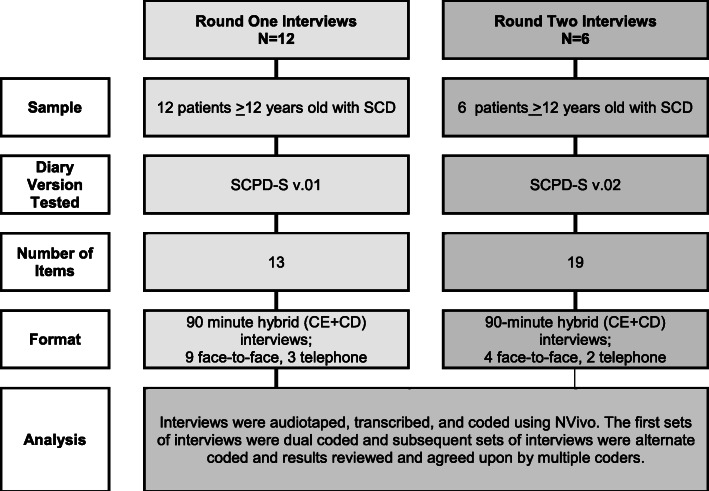
Description of Study Methodology, Divided by Interview Round. Abbreviations: SCD = sickle cell disease; SCPD-S = Sickle-Cell Pain Diary- Self Report; CE = concept elicitation; CD = cognitive debriefing

**Fig. 2:**
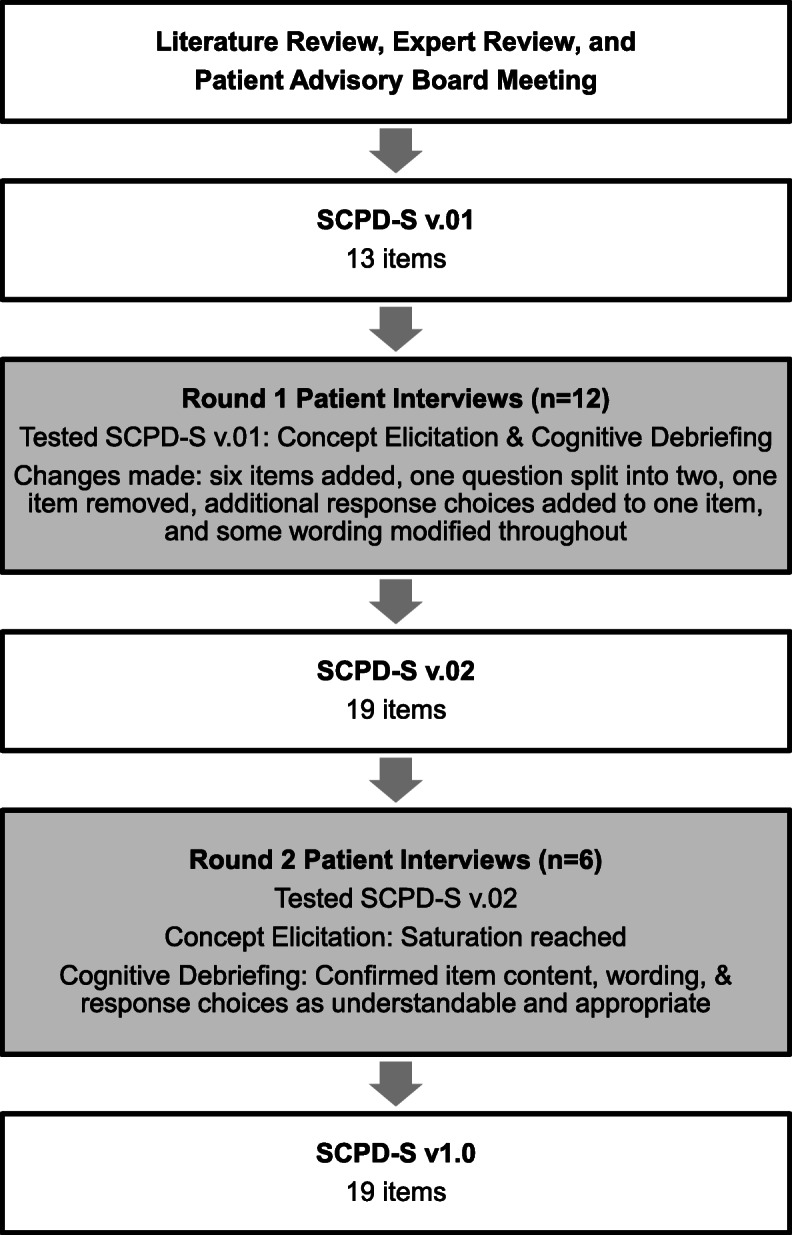
Revision History of the Sickle-Cell Pain Diary- Self Report (SCPD-S)

An overview of the methods used for each type of interview is presented in Fig. 1 and the revision history of the SCPD-S is presented in Fig. 2. Interviews were conducted in two rounds in multiple US locations and via telephone. All interviews were completed by researchers with extensive experience and training in conducting qualitative interviews.

In round 1, hybrid interviews were conducted that included both concept elicitation and cognitive debriefing methods. In the concept elicitation segment of the interview, patients were asked about their daily experiences with SCD symptoms and the impact of those symptoms on aspects of their lives such as work, school, relationships, and social and recreational activities. They were then asked to discuss symptoms experienced during a SCPC, what a typical day looks like both during and outside of a crisis, and what treatment methods they seek when experiencing a crisis. This segment of the interview served to understand which concepts were important to include in the SCPD-S.

A cognitive debriefing approach was used in the second segment of the interview, during which each element of the diary (instructions, recall period, items, response sets, and skip patterns) was tested to evaluate the relevance and comprehensibility of the SCPD-S. The cognitive debriefing portion used a ‘think-aloud’ process: patients were asked to complete the SCPD-S while verbalizing their thoughts about how they answered each question [19]. Patients were then asked to describe any aspects of the diary that they found challenging or confusing. Next, the interviewer probed areas that appeared to be confusing based on patients’ facial expressions, pauses, or comments. Finally, patients were asked a set of structured queries to ensure that all elements of the daily diary were addressed.

The SCPD-S v.01 was revised based on patient feedback from round 1 interviews, resulting in v.02. Round 2 interviews used the same methodology as round 1 (hybrid concept elicitation and cognitive debriefing), with different patients. The SCPD-S v.02 was subsequently revised based on patient feedback from round 2 interviews, resulting in SCPD-S v1.0.

### Data analysis

After transcription of audio-recordings, data were coded and analyzed using identical methodology for both rounds of interviews. Concept elicitation data were coded using NVivo software and analyzed using a content thematic analysis method [20]. This strategy is in accordance with the principles of grounded theory, an inductive methodology whereby concepts emerge from patients rather than researchers imposing an a priori hypothesis [21].

An analysis of saturation was also conducted on data from the concept elicitation portion of the interviews. Saturation is the point at which no new relevant information emerges, and provides evidence that enough interviews were completed to fully understand concepts important to patients. This strategy allows researchers to determine if new concepts are elicited as data analysis progresses, and can provide assurance that enough interviews have been conducted to fully capture the range of patient experiences [21–23]. Data were separated into groups of three interviews and an iterative approach was used for the evaluation of saturation, whereby initial interviews were analyzed and compared with subsequent interviews.

For the cognitive debriefing segment of the interviews, narrative data were coded using a series of ratings to systematically summarize feedback. Transcripts were reviewed and coded for content related to each element of the daily diary to determine whether patients found each element to be relevant and understandable. Coding was completed using NVivo software.

## Results

Eighteen total interviews were conducted in two rounds. Results from the concept elicitation portions of all interviews (rounds 1 and 2 combined) are presented first. These results are separated to discuss concepts related to SCD in general first, and then present concepts related to SCPCs.

Results from the cognitive debriefing portions of interviews are separated into findings from each round of testing because patients responded to different versions of the diary in each round.

### Patient characteristics

Twelve round 1 interviews were completed in-person in two US locations (*n* = 9, 75%) and via telephone (*n* = 3, 25%).^1^ Round 2 interviews were conducted in-person in one location (*n* = 4, 67%) and via telephone (*n* = 2, 33%). Patients ranged from 12 to 50 years old (M = 29 years), and 72% (*n* = 13) of all patients were male. One patient in round 1 (8%) self-reported being of Hispanic descent, and all others across both rounds (*n* = 17 patients (92%) were of African American descent. Almost half of the patients had a ‘less than high school’ education level (*n* = 8, 44%); however, all 8 of these participants were 18 years or younger, indicating that they were still attending high school at the time of the interview. Five participants (28%) reported that they seek out clinical care every time or almost every time they experience an SCPC (Table 1).

**Table 1:** Patient characteristics

Patients	Round 1 Hybrid Interviews SCPD-S v0.1 *n* = 12	Round 2 Hybrid Interviews SCPD-S v0.2 *n* = 6	Total Sample *n* = 18
**Age**			
Mean (SD)	33 (15.5)	20.5 (9.8)	28.8 (14.8)
Range	12–50	13–40	12–50
**Gender**	*n (%)*	*n (%)*	*n (%)*
Male	10 (83.3%)	3 (50%)	13 (72.2%)
Female	2 (16.7%)	3 (50%)	5 (27.8%)
**Education**			
< High school	4 (33.3%)	4 (66.7%)	8 (44.4%)
High school/GED	1 (8.3%)	–	1 (5.6%)
Some college	1 (8.3%)	2 (33.3%)	3 (16.7%)
Associate’s degree	2 (16.7%)	–	2 (11.1%)
Bachelor’s degree	4 (33.3%)	–	4 (22.2%)
Post-graduate degree	–	–	–
**Race**			
African-American	11 (91.7%)	6 (100%)	17 (94.4%)
Hispanic	1 (8.3%)	–	1 (0.55%)
**US Region of Residence**			
Northeast	4 (33.3%)	–	4 (22.2%)
Pacific	1 (8.3%)	–	1 (5.6%)
Southeast	7 (58.3%)	1 (16.7%)	8 (44.4%)
Midwest	–	5 (83.3%)	5 (27.8%)
**# of SCPCs in the past 12 months**			
Mean (SD)	6.2 (4.4)	8.2 (6.8)	6.8 (5.2)
Range	1–16	1–20	1–20
**Reported healthcare utilization during SCPC**			
Every time or almost every time	3 (25%)	2 (33.3%)	5 (27.8%)
Sometimes but not all of the time	7 (58.3%)	4 (66.7%)	11 (61.1%)
Treat at home/see regular doctor	2 (16.7%)	–	2 (11.1%)

### Concept elicitation: sickle cell disease

#### Symptoms and impacts related to SCD

The two most frequently reported symptoms were pain (*n* = 14, 78%) and fatigue (*n* = 11, 61%). Other commonly reported symptoms included: joint problems/stiffness (*n* = 6, 33%), shortness of breath (*n* = 4, 22%), and stress, headaches/migraines, and jaundice (all *n* = 3, 17%).

Patients talked about how their daily lives were affected by SCD, with most (*n* = 14, 78%) describing considerable impacts from daily SCD symptoms. Some impacts were the result of limitations imposed by symptoms, while others were the result of self-imposed limitations due to fear of triggering a pain crisis. Six areas of impact were described during interviews: social /recreational activities (*n* = 15, 83%), activities of daily living (such as dressing or grooming; *n* = 12, 67%), physical functioning (n = 12, 67%), work/school (n = 12, 67%), emotional health (*n* = 11, 61%), and relationships (*n* = 8, 44%). Table 2 provides quotes from patients related to daily impacts.

**Table 2:** Example Patient Quotes: Daily Impacts of Sickle Cell Disease (SCD)

Area of Impact	n (%)	Representative Patient Quote
Social & Recreational	15 (83%)	I love people, I like to go out and do all different types of things but it does have an impact. It’s kind of hard for me to actually plan things in advance. We used to do that but now it’s like the plans are made at the last minute versus two month from now we’re going to go here because it seems like every time I make plans to go somewhere then I get a pain crisis. Not all the time so I’m the one- I try to find the deals and everything, reserve everything like two months away but I don’t do that now. I make the plans that week or that week before and just pray that everything works out. (002)
Activities of Daily Living	12 (67%)	Well, usually if I’m doing housework, say cleaning, I can’t do it all. I have to— I get shortness of breath. So, if I’m cleaning up, mopping, I’ll mop and then I have to take a breath, sit down for a few minutes and then I can do some more. Because I’ll get tired and have to stop and rest for a few and then I can go back and do some more. (012)
Physical Functioning	12 (67%)	I like to play basketball, but it just prevents me from doing that. Sometimes I just want to stay active, but [SCD] just gives me less energy to, and that just prevents me from staying active. (013)
Work & School	12 (67%)	I’ve worked in the past. They were like little or minor three- to four-hour jobs. I’ve been getting sick for too long. Job has told me, “Well, we can’t accommodate you anymore” because I’m absent frequently. (011) When I’m having pain and I do go to school, it distracts my―like I can’t concentrate like on the school lesson as much. I get a lot of absences, which also kind of affects my grades. It’s hard to take tests after the lesson has been taught when you weren’t there. (018)
Emotional Health	11 (61%)	I’m not surprised that anyone that I know that has sickle cell doesn’t get depressed. You’re always wondering if I’m going to live another day. (014)
Relationships	8 (44%)	As far as relationships, I guess some people may not understand. It’s kind of frustrating but now I don’t let it bother me. When you say, “I’m hurting” they don’t really know. I told them if you haven’t really been a victim of pain and I’m just saying I’m hurting wherever, you can’t relate because you don’t know. It’s always like “Really, you’re hurting like that?” I’m like, “Yes. I wouldn’t say it if it wasn’t.” (002)

#### Impacts on social and recreational activities related to SCD

Impacts on social and recreational activities were reported by 15 patients (83%). Patients described avoiding or making accommodations for activities due to symptoms or fear of triggering a pain crisis. In particular, seasonal conditions such as the weather or temperature fluctuations were reported as a major determinant of whether or not patients decided to leave their home for activities. Travel was also reportedly difficult due to concerns that the air pressure in airplanes could worsen symptoms and sitting for long periods of time could be painful.

#### Impacts on activities of daily living, physical functioning, work and school related to SCD

Many patients (*n* = 12; 67%) discussed impacts on activities of daily living, including getting dressed, going to the bathroom, running errands, and lifting heavy objects. Housework in particular required either assistance from others or modifications. Adolescent patients (aged 12–17) specifically noted limitations on physical functioning, as many were unable to participate in common school activities such as extracurricular sports.

Work and school activities were also significantly impacted by SCD. Half of all adult patients were not currently working due to SCD-related problems. Reasons given for leaving the workforce included stress, physical problems, and taking too much time out of work due to SCD. Adults who were currently working all reported work-related limitations and necessary modifications.

Adolescents noted an impact on school performance. In particular, two adolescent patients discussed getting behind on schoolwork due to difficulty concentrating and missed classes.

#### Impacts on emotional health and social relationships related to SCD

Patients reported that SCD took a toll on their emotional health and well-being (*n* = 12; 67%). Commonly reported emotional difficulties were sadness, anxiety, and stress. Emotional impacts were typically related to limitations they experienced due to SCD, uncertainty about the future, and helplessness in dealing with symptoms and impacts. Patients also reported impacts on their relationships. Some patients (*n* = 7, 39%) reported having one or more close friends or family members who “didn’t understand” the disease and either distanced themselves or shamed patients for needing accommodations.

### Concept elicitation: sickle cell pain crises

#### Symptoms and impacts related to SCPCs

Patients described the pain sensation during an SCPC, using descriptors such as ‘sharp,’ ‘stabbing,’ ‘throbbing,’ ‘burning,’ ‘grinding,’ and ‘hammering.’ Patients’ experiences of pain crises varied both within and across individuals. Some reported that it was more typical to have frequent, intense, and long lasting SCPCs, and some reported that it was more typical to experience occasional, milder, and shorter crises. Patients reported experiencing a median of 5 SCPCs per year, with responses ranging from “1 - 4 per year” to as often as every 2 weeks. Across patients, the shortest SCPC lasted only a few hours and the longest crisis experienced lasted 8 weeks. Some individuals reported a tendency to experience longer crises and others reported having shorter crises, but all experienced variability in their own SCPC experiences. While some reported crises happening “out of nowhere,” others reported precursor symptoms that alerted them to the oncoming SCPC. Patients described multiple triggers believed to cause their SCPC.

SCPCs impacted many areas of life, including work and school (*n* = 16, 89%), emotional health (*n* = 15, 83%), sleep (*n* = 15, 83%), medication use (*n* = 14, 78%), physical functioning (*n* = 13, 72%), and activities of daily living (*n* = 13, 72%; Table 3).

**Table 3:** Example Patients Quotes: Sickle Cell Pain Crise is (SCPC)

Symptoms of SCPC	n (%)	Representative Patient Quotes
Pain	14 (78%)	Sometimes I get a slight feverish feeling, then I start getting these little pins and needle pains that start out. Usually it’s isolated, or sometimes it moves up to my whole leg, or part of my leg. I pay attention to, “Oh God. I pray it doesn’t spread all over.” Usually if it’s an isolated thing, I pay attention to those things. I do notice I get feverish. I get sharp pains. Extremely tired. I’m out of breath. I just get extremely weak. My oxygen levels are low, so I have to drink water. I’m not thirsty, but I have to drink it. I just feel like crap. You just know when it’s going to happen. It creeps up on you. (014)
Fatigue	11 (61%)	
**Impacts of SCPC**	**n (%)**	**Representative Patient Quotes**
Work & School	16 (89%)	I have worked through a crisis before. Because sometimes I’m OK in the morning. I feel a little tired and sluggish but, I’m like, “I’m not going to call out.” And then I would go in and then after I’ve been working for a couple of hours, I’m like, “Oh, this today.” And it would start sometimes in my back and I would be like, “Oh, God.” And it’s so busy and I can’t just leave. So, I’m just sitting there working through it and I’m taking something strong but, not Oxycodone because you can’t take that at work and I’ll be sitting there dozing off. So, I’ll take something and just keep drinking Gatorade because that’s the only thing I can do. (012)
Emotional Health	15 (83%)	Well, emotionally it’s like, “Why is this happening?” I feel like I just sometimes I just want to cry. I can’t deal with this. I don’t know why this is happening. I thought I did everything I was supposed to do. And it’s still here anyway. But, what can I do? It makes you sad. But, what can you do? You just try to fight through it. (012)
Sleep	15 (83%)	If I’m in pain, sometimes it’s difficult to fall asleep because the pain is so intense. Sometimes, depending on the pain medication I take it will keep me up. Like I take Percocet and I’ll fall asleep for maybe two to three hours and then I’m up again. With the medicine that they give me in the hospital, I’m always just in and out of it. I might be awake for a period of time and just nod off. (001)
Medication Use	14 (78%)	If I am having a crisis there a medications I have to increase, then there’s going to be medications that I’m going to have to take as well that I don’t take regularly. (002)
Physical Functioning	13 (72%)	Pain crises can be paralyzing at time. A minor pain crisis, I am good, I can move around, and be a little slow, but I can move around, but in a severe pain crisis, it’s dying. (003)
Activities of Daily Living	13 (72%)	Pain crisis, a mild one, I would say allows me to do the normal activities. I would consider that a pain of, for me, anything less than a 7. So, on a scale of 1 to 10, anything less than for me personally 7, I can still kind of function. I can take pain medication as far as like an ibuprofen or a Bayer, and it still allows me to do basic functions. (011)

#### Impacts of SCPCs on work and school, activities of daily living, and physical functioning

Most patients who were either currently employed or had been employed in the past reported having missed work due to an SCPC, and also having attended work during crises so they would not miss more days of work. Patients who attended work with an SCPC typically did not do so during the peak of pain, but still described attempting to “push through” the workday with accommodations such as modified duties, taking breaks, and consuming food/water. Most adolescent patients reported not attending school during a crisis – either staying home, or, if the crisis happened while attending classes, leaving early. Once an intense crisis was at a peak, the effects were described as debilitating across all patients. In these cases, patients typically reported confining themselves to a bed or couch all day and resting or, if the crisis lasted a particularly extended length of time, patients reported going to the hospital.

#### Impacts on emotional health, sleep, and medication use related to SCPCs

Patients reported experiencing effects on emotional health (*n* = 15, 83%), including stress, anxiety, and helplessness when experiencing an SCPC related to the pain and uncertainty about how long the crisis would last. Patients described difficulty sleeping during an SCPC due to the pain (*n* = 15, 83%). In some cases, pain medication was reported to improve sleep; other patients said that pain medication interfered with sleep. Most patients reported sometimes increasing the dose, frequency and types of medications they take when experiencing an SCPC (*n* = 14, 78%).

### Concept elicitation: healthcare utilization during an SCPC

Only a small number of patients (*n* = 5, 28%) reported ultimately seeking care outside of the home for most pain crises. For these patients, seeking care was considered to be a last resort after the pain had reached a level (on a scale of 0–10) of “ten and higher.” Many patients reported distress related to making decisions about whether and when to go to the hospital. Patients reported using a variety of pain crisis prevention and minimization strategies to delay having to seek care outside the home for as long as possible. Strategies included: rest, hydration, breathing exercises, applying a heating pad, music, meditation, and increased medication use. Of the subset of patients who shared reasons for delaying seeking treatment outside the home, the most commonly reported reasons included: feeling stigmatized by doctors and other treatment professionals or not being believed about pain levels (*n* = 7, 39%) and long wait times in emergency rooms (*n* = 5, 28%; Table 4).

**Table 4:** Example Patient Quotes: Healthcare Utilization during SCPC

Reason for Non-utilization of Healthcare for SCPC	n (%)	Representative Patient Quotes
Stigmatization by healthcare professionals	7 (39%)	Sickle cell patients, we have big issues with the hospital because we get accused of drug seeking discrimination. When you go, you’re supposed to get treated immediately. Sometimes we’re waiting in the emergency room for six hours before you can get pain medication. So, if you kind of feel a pain crisis coming on, you’re taking your medicine at home but that doesn’t work. So, now, you’re going to the ER. But now, I have to wait six hours before I can get pain medication…So, once that happens for us too many times, we can be in the worst pain. And we rather sit home and attempt to treat it at home first before we want to deal with any of that. (011) Sometimes they’re like, “Well you’re having a crisis.” “Yeah, I know. That’s why I’m here.” And it’s like, “Well, how do you feel?” And to me it’s always 10. If I’m in there it’s because I can’t control it anymore, or I’ve taken everything I can take. (021)
Long wait times at hospital	5 (28%)	
Ineffective treatment options	4 (22%)	
Lack of clinician knowledge on SCPC	3 (17%)	
Racial discrimination	2 (11%)	

### Concept elicitation: addition of new concepts

The findings from round 1 concept elicitation interviews resulted in the addition of six items, three of which were designed to assess an SCPC, and three of which were designed to assess impacts of SCD in the absence of an SCPC. One existing item related to SCPC impacts was split into two items to better reflect the experiences of patients. The revised version of the diary, which incorporated these additions, was named SCPD-S v.02. No new concepts emerged during round 2 concept elicitation interviews, and all of the added concepts were confirmed.

Multiple concepts emerged regarding patients’ experience of SCPCs that necessitated additions to the SCPD-S v0.1. First, because half of the patients in round 1 discussed emotional difficulties (*n* = 6, 50%), an item was added to assess the degree to which an SCPC resulted in such difficulties. Second, patients clearly reported that they experienced impacts on activities of daily living and social/recreational activities, and that these impacts are distinct from one another. As a result, one item of the SCPD-S v.01 that asked about general activities was split into two items (one on activities of daily living and one on social and recreational activities) to provide better and more precise coverage of these concepts. Third, two items were added because patients indicated the importance of the duration of SCPCs and healthcare utilization as a result of SCPCs.

Fourth, although the SCPD-S was developed specifically to assess the frequency, duration, and impacts of SCPCs, results of round 1 concept elicitation interviews provided evidence to suggest that additional items should be added to assess patients who experience SCD-related pain that is not due to an SCPC. Because patients discussed experiencing fatigue, difficulty sleeping, and emotional difficulties on a daily basis, even when not experiencing an SCPC, three additional items were added to the SCPD-S to assess these impacts in patients who had not experienced an SCPC in the past 24 h (Table 5).

**Table 5:** Samples of items added to the SCPD-S v.01

Item added to SCPD-S v.01	Rationale for Addition	Patient Quotes (on tested item)
How much emotional difficulty did you experience due to your sickle cell pain crisis during the past 24 hours (for example, stress, anxiety, sadness, and depression)?	Five out of 12 patients reported that mental and emotional health were important concepts to ask about in a daily diary on sickle cell pain and crises.	You could go with the emotional aspect as well, were you able to—I don’t, I guess I don’t know, maybe if I thought about my emotions at that time but I probably don’t…but I think maybe just even seeing where a person’s state of mind is? Either in or outside of the crisis, might help reveal… some information, I guess, if you were to pose questions towards your overall state of mind. (007)
Did the pain crisis last all of the past 24 hours or less than the past 24 hours?	Seven out of 12 patients expressed a preference for a question that would capture whether the sickle cell pain crisis had lasted more than 24 h so that they could skip filling out the number of hours under those circumstances.	We’re in pain so much we just don’t keep track of the time. (001)

### Concept elicitation: saturation

Saturation analyses revealed that minimal new concepts were added through later code sets, an indication that saturation was reached (Table 6). Specifically, in round one, 44 concepts were identified and labeled for coding; rounds 2 and 3 added 8 new codes each and rounds 4–6 each had between 0 and 2 new codes.

**Table 6:** Saturation Grid

	Number of New Concepts Emerging by Theme					
Code Set #; Interviews	Code set 1;	Code set 2;	Code set 3;	Code set 4;	Code set 5;	Code set 6;
	1–3	4–6	7–9	10–12	13–15	16–18
	Round 1 Interviews				Round 2 Interviews	
**Major Theme**						
SCD symptoms	10	3	4	–	–	1
SCD impacts	9	1	–	–	–	–
SCPC symptoms	9	3	3	–	1	1
SCPC impacts	10	1	1	–	–	–
Treatment Decisions	6	–	–	–	–	–
Total	44	8	8	0	1	2

### Cognitive debriefing

#### Round 1 (*n* = 12)

Round 1 of interviews tested the SCPD-S v.01 which consisted of 13 total items with 12 participants. Overall, the SPCD-S was generally found to be easy to understand and complete. Also, patients were relieved to hear the finalized SCPD-S would be completed electronically (on a tablet, or smartphone) and they would not have to read and follow the skip pattern. Instructions were revised after round 1 to increase clarity. The recall period was endorsed as appropriate by most (*n* = 11, 92%) patients in round 1.

Eight of the items were found to be easy to understand and relevant to patient experiences with SCPCs. Modifications were made to 5 items, and one item was removed. The removed item, ‘Did you have pain today?’ was well understood by some patients (*n* = 7, 58%), but others found it difficult to distinguish sickle cell pain that was not crisis related from crisis-related pain. In general, the modifications made were minor wording improvements for clarity. However, misunderstandings on one item related to the duration of the pain crisis within the past 24 h required more significant modification.

Response options were rated as clear and easy to understand by patients in 11 of the 13 items. Response choice sets from 3 items were changed as well (see Table 7 for examples of changes made between v.01 and v.02 of the SCPD-S).

**Table 7:** Samples of items and response choices modified in the SCPD-S v.01

Tested Item or Response Choice (SCPD-S v0.1)	Rationale for Modification	Patient Quotes (On Tested Item or Response Choice)	Final Modified Item(s) or Response Choice(s) (SCPD-S v1.0)
**Item** How long did the pain crisis episode last in total during the last 24 hours? Select the time point that best represents the total duration (in hours and/or minutes). If the pain crisis episode lasted for more than 24 hours, please report 24 hours and 00 minutes.	Patients reported problems with recording exact times for the duration of their pain crises, therefore, the item was changed to ask for an approximation.	That was more like an approximate amount. Most of the time I don’t really keep track of time. (001)	Approximately how many hours did the pain crisis last during the past 24 hours?
**Response Choice** How long did the pain crisis episode last in total during the last 24 hours? Select the time point that best represents the total duration (in hours and/or minutes). If the pain crisis episode lasted for more than 24 hours, please report 24 hours and 00 minutes. □□ Hours Minutes	Eleven out of 12 patients expressed a preference for only recording hours rather than both hours and minutes.	This is kind of lengthy. (002) [*Interviewer: If we did it that way, is it necessary to have the minutes at all or should we just say the number of hours if it’s less than a day?]* I would say hours. (002)	Item response choice: □□ Hours
**Item** During the last 24 hours, did you take any medication to control your pain?	When probed, multiple patients reported not including over-the-counter medications or medications prescribed while in the hospital when answering the question.	I think it was asking about the medication…which, in my head, was the medication that I gave myself. (003)	Did you take any medication to control your pain, including prescribed and over-the-counter medication, during the past 24 hours?
**Item** How much fatigue did you experience due to your sickle cell pain crisis during the last 24 hours?	Ten out of 12 patients endorsed and understood this item. Two adolescent interviewees did not understand what was meant by fatigue, so definitions and descriptors were added for clarity.	I didn’t really understand the word fatigued, but you told me. (013)	How much fatigue did you experience due to your sickle cell pain crisis during the past 24 hours (for example, tiredness, very low energy, having trouble keeping your eyes open, or frequent napping)?

#### Summary of changes made to the diary before round 2

In summary, based on the concept elicitation and cognitive debriefing results from round 1, the following changes were made (see Table 7 for examples):

• 3 items were added to assess impacts in patients who had not experienced an SCPC in the past 24 h

• 3 items were added to address new concepts

• One question was split into two questions

• Additional response choices were added to one response set based on cognitive debriefing from round 1

• 5 items were modified to improve wording

• 1 item was removed

• The total # of questions was increased from 13 to 19

#### Round 2 (*n* = 6)

After revision based on results from round 1, the SCPD-S v.02 consisted of 19 items and was tested in round 2. Overall, patients found the SCPD-S easy to understand and complete. Instructions were confirmed as comprehensible. All patients (n = 6, 100%) agreed that the recall period was appropriate. Patients found the wording of items to be clear and well-written. One item was changed to include the addition of ‘specialist’ to a list of providers a patient might visit with during an emergency. Otherwise, no changes were made to items. Items were endorsed as relevant and patients confirmed the good fit of the response options. Patients endorsed the comprehensiveness of the diary and no additional items were added.

In sum, cognitive debriefing of round 2 interviews further confirmed the relevance of the concepts measured in the SCPD-S, and the comprehensibility of the instructions, recall period, items, response sets, and skip patterns for patients with SCD.

After edits from round 2 and additional minor changes as a result of proofreading and to improve adaptability to an electronic format, the SCPD-S version number was changed to 1.0, as a developed version that has evidence of content validity for clinical and research use with patients who have SCD and experience SCPCs. Contents of SCPD-S v1.0 are summarized in Table 8. This version will be used in future studies and evaluated for psychometric reliability and validity.

**Table 8:** Summary of contents of the SCPD-S

Item Content	Number of Items
Items Related to SCPC (only completed if as SCPC was experienced in the past 24 h)	
Observed signs of SCPC	1 item
Duration of SCPC	2 items
Level of pain related to SCPC	2 items
Healthcare utilization due to SCPC	1 item
Medication use due to SCPC	2 items
*Impact of SCPC on the following domains:*	
Work or school	2 items
Activities of daily living	1 item
Impact on social and recreational activities	1 item
Sleep	1 item
Fatigue	1 item
Emotional difficulty	1 item
Items Unrelated to SCPC (only completed if no SCPC experienced in past 24 h)	
Signs of pain outside of an SCPC	1 item
Interference of SCD with sleep	1 item
Impact of SCD on fatigue	1 item
Impact of SCD on emotional difficulty	1 item

Abbreviations: *SCD* sickle cell disease, *SCPC* sickle cell pain crisis, *SCPD-S* Sickle-Cell Pain Diary-Self Report

## Discussion

Patients in this study described the immense impact SCD and SCPCs have on their health-related quality of life. SCD significantly impacted activities of daily living, work and school, and emotional health. SCPCs greatly affected sleep, medication use, and physical functioning. With this data as the backbone of important aspects for a PRO measure for patients with SCD to be used in clinical trials, other research studies, and as part of clinical practice, we significantly expanded the SCPD-S after the first round of interviews.

The SCPD-S underwent cognitive debriefing testing in two rounds of patient interviews, and the diary was revised after each round, so that the version reviewed in the second round incorporated feedback from the first round of interviews. Patients confirmed that the concepts in the SCPD-S are relevant to people who have SCD and experience SCPCs. The revised SCPD-S v.02 that was tested in the second round was well-understood by patients. Generally, the SCPD-S was regarded as appropriate, comprehensive, and true to patient experiences. The changes to the SCPD-S as a result of patient feedback highlight the importance of the content validation process when developing a PRO measure and of re-testing a PRO after modifications have been made [24]. Had the study ended after the first round of interviews, the opportunity to evaluate how changes made after the first round of interviews impacted content validity would have been missed. The use of an iterative approach follows best practice guidelines of contemporary validity testing theory standards, which impresses that validity goes beyond a single content validation study or examination of psychometric properties, and instead requires the ongoing accumulation and evaluation of sources of evidence, taking into account the purpose of the PRO measure and eventual interpretive framework [23, 24].

The SCPD-S was developed to fill three primary gaps in the ability to measure the patient experience and impact of SCPCs on patients’ health-related quality of life: (1) that research studies and clinical practice tend to rely heavily on capturing data on SCPCs requiring healthcare utilization (e.g. emergency room or hospital admissions), which does not fully capture burden of illness and misses SCPCs that are treated at home; (2) that SCPCs are of variable duration and may be associated with health-related quality of life impacts pre- and post- the acute phase requiring more granular data capture; and (3) no other daily diaries had been developed with a clear focus on the health-related quality of life of a patient before, during, after and between SCPC events. Existing PRO measures used longer recall periods (such as past week or past month) that required patients to consider their average experience during an SCPC, despite there being tremendous variation within individual (across separate pain crises) and across individual SCPCs. Further, the existing measures were not focused on measuring what would happen if the frequency of SCPCs changed. Developing the SCPD-S with a past 24 h recall period provides a significant improvement in the ability to measure the depth and breadth of patient experience including capturing at home SCPC care; critical for better understanding potential need for change in clinical care, and for research studies. Patients may also find it useful to use the SCPD-S on their smartphone, tablet, or computer to improve communications with their treatment providers about their experiences.

Study limitations include only using one mode of administration (paper). In addition, our sample was 72% male yet approximately 50% of SCD patients are female [25], and while our sample included individuals as young as age 12, the oldest patient was 50. In addition, our sample included primarily African-American participants (94%). While SCD is found primarily among African-Americans in the US, it does occur in other populations. Thus, future studies should aim to include a greater percentage of female patients, patients older than age 50, include some patients who are not African-American, and include usability testing of the programmed survey.

Planned future work on the SCPD-S includes development of interpretation guidelines for the SCPD-S, including a scoring algorithm and user’s manual. In addition, a research study is underway to examine tests of psychometric reliability and validity of the SCPD-S. Use of the SCPD-S is planned for use in future clinical trials enrolling patients who experience SCPCs as a way to capture the frequency, duration, severity, and health-related quality of life impacts of SCPCs whether the SCPC was treated at home or in an HCP office, SCD clinic, or an emergency room or hospital. Collecting more precise data on a daily basis may help to better define and measure clinically meaningful improvement as a result of treatment in an SCD population. The findings of this study provide evidence of content validity of the SCPD-S.

## Conclusions

This study provided evidence to support the content validity of the SCPD-S, a self-report daily diary. Evidence gathered during two rounds of hybrid, individual, qualitative patient interviews indicated that the SCPD-S is a fit for purpose measure of SCPC-related pain frequency, severity, and impact on health-related quality of life, including fatigue and emotional health. The numerous changes to the SCPD-S as a result of the study findings highlight the importance of the content validation process when developing a PRO measure.

## Data Availability

The data sets analyzed in the current study are available from the corresponding author on reasonable request.

## Footnotes

1 19 interviews were conducted; 1 interview from round 1 excluded due to low patient comprehension.
